# The cypsela (achene) of *Echinacea purpurea* as a diffusion unit of a community of microorganisms

**DOI:** 10.1007/s00253-021-11212-2

**Published:** 2021-03-09

**Authors:** Massimiliano Cardinale, Marian Viola, Elisangela Miceli, Teresa Faddetta, Anna Maria Puglia, Valentina Maggini, Corrado Tani, Fabio Firenzuoli, Silvia Schiff, Patrizia Bogani, Renato Fani, Alessio Papini

**Affiliations:** 1 Department of Biological and Environmental Sciences and Technologies, University of Salento, P.le Lecce-Monteroni, 73100 Lecce, Italy; 2 Institute of Applied Microbiology, Research Center for BioSystems, Land Use, and Nutrition (IFZ), Justus-Liebig-University Giessen, Heinrich-Buff-Ring 26-32, 35392 Giessen, Germany; 3 Laboratory of Biomorphologies, Department of Biology, University of Florence, Via Madonna del Piano 6, 50019 Sesto Fiorentino, Italy; 4 Laboratory of Microbial and Molecular evolution, Department of Biology, University of Florence, Via Madonna del Piano 6, 50019 Sesto Fiorentino, Italy; 5 Laboratory of Molecular Microbiology and Biotechnology, STEBICEF Department, University of Palermo, Viale delle Scienze Ed. 16, 90128 Palermo, Italy; 6 Referring Center for Phytotherapy, Tuscany Region, Careggi University Hospital, Largo Brambilla 3, 50134 Florence, Italy; 7 Department of Experimental and Clinical Medicine, University of Florence, Largo Brambilla 3, 50134 Florence, Italy; 8 Laboratory of Plant Genetics, Department of Biology, University of Florence, Via Madonna del Piano 6, 50019 Sesto Fiorentino, Italy

**Keywords:** *Echinacea*, *Echinacea purpurea*, Endophytic bacteria, Fungi, Anatomy, Cypsela, Perianth

## Abstract

*Echinacea purpurea* is a plant cultivated worldwide for its pharmaceutical properties, mainly related to the stimulation of the immune system in the treatment of respiratory infections. The cypselas (fruits) of *E. purpurea* were examined in order to investigate the presence, localization and potential function(s) of endophytic microorganisms. Electron and confocal microscopy observations showed that three different components of microorganisms were associated to cypselas of *E. purpurea*: (i) one endocellular bacterial component in the cotyledons, enclosed within the host membrane; (ii) another more generic bacterial component adhering to the external side of the perianth; and (iii) a fungal component inside the porous layer of the perianth, the woody and porous modified residual of the flower, in the form of numerous hyphae able to cross the wall between adjacent cells. Isolated bacteria were affiliated to the genera *Paenibacillus*, *Pantoea*, and *Sanguibacter*. Plate tests showed a general resistance to six different antibiotics and also to an antimicrobial-producing *Rheinheimera* sp. test strain. Finally, microbiome-deprived *E. purpurea* seeds showed a reduced ability to germinate, suggesting an active role of the microbiome in the plant vitality. Our results suggest that the endophytic bacterial community of *E. purpurea*, previously found in roots and stem/leaves, might be already carried at the seed stage, hosted by the cotyledons. A further microbial fungal component is transported together with the seed in the perianth of the cypsela, whose remarkable structure may be considered as an adaptation for fungal transportation, and could influence the capability of the seed to germinate in the soil.

Key Points

• *The fruit of Echinacea purpurea contains fungi not causing any damage to the plant.*

• *The seed cotyledons contain endocellular bacteria.*

• *Seed/fruit deprived of the microbiome showed a reduced ability to germinate.*

**Supplementary Information:**

The online version contains supplementary material available at 10.1007/s00253-021-11212-2.

## Introduction

*Echinacea purpurea* (L.) Moench (*Asteraceae*) is a widely cultivated plant, known worldwide for its pharmaceutical properties. This *Echinacea* species (as other species of the same genus) was used as a medicine by American indigenous people in Mexico for the ailment of various diseases, mainly sore mouth and throat, colic, stomach cramps, and toothache (Shemluck 1982). The fruits of *Echinacea* are called cypselas or cypselae, similar to the achenes, but derived from an inferior ovary (Simpson 2006). For this reason, the cypselas, externally to the pericarp present a further structure, the perianth, derived from the flower corolla (Spjut 1994).

The beneficial properties of *Echinacea* are mainly related to the stimulation of the immune system (Stuart and Wills 2003), but also analgesic, anti-inflammatory, and antibiotic activities have been proposed (Parsons et al. 2018). These medicinal effects are attributed to phytochemical compounds such as alkylamides, polysaccharides, and various phenolics like echinacoside, cichoric acid, caftaric, and chlorogenic acid (Parsons et al. 2018; Sharifi-Rad et al. 2018 and references therein).

Miller et al. (2012) and Chiellini et al. (2014) proposed that at least part of these medicinal properties of the plants may depend on the bacterial endophytes and our recent findings suggest that the bacterial endophytes could really affect the therapeutic features of these important medicinal plants (Maggini et al. 2017). Moreover, bacteria from different plant compartments showed specific antibiotic resistance and antibiotic production, suggesting that the bacterial communities may actively select their neighbors in the different plant compartments (Maggini et al. 2018). Further studies about the presence of microbial endophytes in *Echinacea* have shown that the bacterial communities vary between the compartments of the same species and between different species (Chiellini et al. 2014), suggesting the existence of a selective pressure responsible for structuring the microbial communities (Maida et al. 2014; Mengoni et al. 2014).

Endophytes can be defined as microorganisms living within the plant tissues with no pathogenic effects (Wilson 1995) and they are widely distributed in plants (Malfanova et al. 2013; Ryan et al. 2008). The presence of endophytes is considered useful for the plants, by mean of several direct and indirect interaction mechanisms, including hormones-mediated stimulation of plant growth, improvement of mineral nutrition, increase of abiotic stress resistance, and defense from phytopathogens (Lugtenberg and Kamilova 2009; Liu et al. 2017a). Modern “omics” technologies have demonstrated that such beneficial interactions depend on specific genetic traits of these microorganisms (Sharma et al. 2020), including genes involved in mineral nutrient metabolisms, antibiotic production/resistance, and sporulation: interestingly, such traits differ between beneficial microbes co-inoculated in plants (Gamez et al. 2020).

In *E. purpurea*, the presence of root and shoot endophytes has recently been related to the increase of alkilamides content and to the higher expression level of the valine decarboxylase (VDC) gene (Maggini et al. 2017), involved in the biosynthesis of the amine moieties of alkylamides (Rizhsky et al. 2016). These compounds, together with other phenolics, have been found at high levels in seeds of *Echinacea* (Parsons et al. 2018). However, no data concerning the presence, biodiversity, and localization of *Echinacea* seed-borne endophytes are known.

Seed-associated endophytes have been described as capable of performing different functions essential for the plant, such as phytohormone production (Shahzad et al. 2017; Alibrandi et al. 2018), seedling and plant growth promotion (Rahman et al. 2018; White et al. 2017; Hardoim et al. 2012), siderophore production (Díaz Herrera et al. 2016; Alibrandi et al. 2018), as well as antifungal property and antibiotic production (Verma et al. 2017; Fürnkranz et al. 2012; Donnarumma et al. 2011). Moreover, it has been recently shown in cereals that seed-associated bacteria have coevolved with the plant hosts during domestication, likely as a result of mutualistic reciprocal advantage (Abdullaeva et al. 2021).

Antibiotic resistance has been previously evaluated for *E. purpurea* and *Echinacea angustifolia* plants-associated bacterial endophytes (Maggini et al. 2018; Mengoni et al. 2014), and it has been hypothesized to be one of the factors shaping the plant-associated bacterial communities. Among seed endophytes, antibiotic resistance could hypothetically be implied in determining communities’ structure leading to the selection of those strains exhibiting higher probability of persistence and vertical transmission to the next plant generation.

The aim of the present work was to analyze the cypselas of *E. purpurea* in order to evaluate the possible presence of fungi and bacteria in the different components of the seed, i.e., perianth, pericarp, and cotyledons, to understand whether the symbiosis develops by secondary contact of the plant tissue with bacteria present in the environment, or whether the cypsela itself shows adaptation related to microbes’ transportation. Additionally, this work aimed at exploring the biodiversity of seed-borne endophytes by extracting and characterizing them from a taxonomic and functional viewpoint. Moreover, since seed-borne endophytes could be related to seed germination capability of different *Echinacea* spp., seed germinability was also evaluated.

## Materials and methods

### Plant material

Seeds of the *E. purpurea* were provided by the “Il Giardino delle Erbe,” an association devoted to the conservation of plant biodiversity located in Casola Valsenio, Italy. The morphological characters were checked by A. P. and allowed identification at the species level. A part of the seeds is conserved at the Dept. of Biology of the Univ. of Florence. The plants are perennial, and are cultivated and maintained by the association “Il Giardino delle Erbe.”

We followed here the nomenclature and the general description of the *Echinacea* cypsela by Parsons et al. (2018) and Schultess et al. (1991). For the identification of the most frequent components of the parenchyma cotyledon cell (oil bodies and protein bodies), we followed Evert (2006), specifically page 54.

### Fixation and embedding

Ten developing seeds were prefixed overnight in 1.25% glutaraldehyde at 4 °C in 0.1 M phosphate buffer (pH 6.8), and then fixed in 1% OsO_4_ in the same buffer for 1 h. After dehydration in an ethanol series 30–100%, 5 min each step, and a propylene oxide step, samples were embedded in Spurr’s epoxy resin.

### Sectioning and staining for light and fluorescence microscopy

Seeds embedded in Spurr’s epoxy resin were transversely sectioned with glass knives to obtain semi thin sections (1–5 μm), which were stained with toluidine blue, 0.1%, then observed and photographed with a Leitz DM RB light microscope (Leica Microsystems, Mannheim, Germany). Not embedded seeds were instead sectioned with a cryostat to generate sections of 10–20 μm of thickness. Some of these seed sections were stained with 1% phloroglucinol (w/v) in 12% HCl for 5 min and observed with a brightfield light microscope for detecting lignin and with periodic acid-Schiff stain (PAS) reaction to detect starch. Another set of cryostat sections were stained with Sudan III for the detection and localization of lipids under brightfield microscopy (Brundrett et al. 1991). The remaining Cryostat sections were stained with Fluorol Yellow 088 and viewed with a fluorescent microscope Leica DM RB Fluo (Leica Microsystems, Mannheim, Germany) in the range of 515–565 nm (green) to detect lipids (Brundrett et al. 1991). The image series with differential staining were treated with the python program ALLAMODA 2.0 (Papini 2012) to reduce noise.

### Transmission electron microscopy

Seeds embedded in Spurr’s epoxy resin were further cut with a diamond knife to generate sections that were approximately 80 nm thick. These sections were stained with uranyl acetate and lead citrate, and then examined with a Philips EM300 TEM (Philips Electron Optics, Eindhoven, The Netherlands) operating at 80 kV.

### Microscopy analysis by fluorescence in situ hybridization–confocal laser scanning microscopy

Seeds of *E. purpurea* were embedded in tissue freezing medium Jung (Leica Instruments GmbH, Nussloch, Germany), and longitudinal cryosections of 30 μm were obtained using the low-temperature constant-cooling cryostat HM 500 OM (MICROM, Walldorf, Germany) at − 20 °C; the cryosections were gently washed in × 1 phosphate-buffered saline (PBS) to remove the embedding medium, and fixed in 3:1(v:v) 4% paraformaldehyde:PBS for 6 h at 4 °C, then washed three times in ice-cold PBS (for 10/20/30 min stepwise, at 4 °C), and finally stored at − 20 °C in 1:1 (v:v) ice-cold PBS:ice-cold 96% ethanol, until FISH staining.

The cryosections were stained by in tube-FISH according to Cardinale et al. (2008), using the Cy3-labeled EUB338MIX probe (the equimolar mixture of EUB338, EUB338II, and EUB338III probes), universal probe for bacteria (Amann et al. 1990; Daims et al. 1999). Hybridization was performed at 42 °C for 2 h in the dark, followed by washing at 43 °C. Stained samples were dipped for 5 s into ice-cold water, placed on a glass slide, dried out with soft compressed air, immediately mounted with antifade reagent, covered with a coverslip, and finally sealed with nail polish. The occurrence of false positive signals derived from aspecific adhesion of FISH probes or fluorochromes to seed structures was checked by staining a subsample with Cy3-labeled NONEUB probe.

Stained samples were observed with the confocal laser scanning system Leica SP8 (Leica Microsystems GmbH, Mannheim, Germany). Four confocal channels were acquired, one for the Cy3-conferred signal of the EUBMIX probe (excitation, 561 nm; emission, 570–625 nm) and three further channels for the autofluorescence of the seed tissues (excitations, 405, 488, and 633 nm; emissions, 420–480, 500–545, and 650–700 nm, respectively). All signals were acquired sequentially. Confocal stacks were acquired with a Leica 63X 1.0 NA water-immersion objective by applying a *Z*-step of 0.6–0.8 μm, and visualized by maximum projections and volume-renderings with the software Imaris version 8.2 (Bitplane, Zurich, Switzerland). Final figures were assembled with Adobe Photoshop CS6 (Adobe Systems Inc., San Jose, USA).

#### Isolation of bacteria from *E. purpurea* seeds

*E. purpurea* seeds (100 mg) were subjected to a step surface sterilization procedure: 3 min wash in sterile distilled water, followed by 1 min in 70% ethanol, 2 min in 2.5% sodium hypochlorite, and two rinses in sterile distilled water.

To confirm that the sterilization process was successful, 1 ml of the last washing water of surface-sterilized seed was incubated on different culture agar media (LB, SFM, R2YE, TSB, PDA) and examined for growth after incubation at 30 °C for 4 days.

The surface-sterilized seeds were immersed in Falcon tubes containing sterile distilled water for 1 h at room temperature and then ground with a Potter-Elvehjem Tissue Grinder (Sigma-Aldrich, St Louis, USA) in 2 ml phosphate buffer saline (PBS: 140 mM NaCl, 3 mM KCl, 10 mM Na_2_HPO_4_, 2 mM KH_2_PO_4_, pH 7.4), and finally shaken at 150 rpm for 1 h. One hundred microliter of suspension were plated on Luria-Bertani (LB), mannitol soya flour (MS) 138, and R2YE agar media. The plates were incubated at 30 °C until appearance of microbial colonies. The colonies, grown on the culture media, were selected by phenotypic criteria (pigmentation and morphology) and repeatedly streaked on new agar media to obtain pure cultures.

#### Random amplified polymorphic DNA analysis

Cell lysates of the endophytic bacterial isolates were obtained by thermal lysis incubating an isolated bacterial colony for each isolate at 95 °C for 10 min, and cooling on ice for 5 min. Amplification of DNA was performed on 2 μl of cell lysate in a 25-μl total volume reaction composed by × 1 reaction buffer, 300 μM MgCl_2_, deoxynucleoside triphosphate (200 μM each), 0.5 U of PolyTaq DNA polymerase (all reagents were from Polymed, Florence, Italy), 500 ng of primer 1253 [5′-GTTTCCGCCC-3′] (Mocali et al. 2003). Amplification conditions were the following: 90 °C for 1 min, and 95 °C for 90 s followed by 45 cycles at 95 °C for 30 s, 36 °C for 1 min, and 75 °C for 2 min. Finally, the reaction mixtures were incubated at 75 °C for 10 min, 60 °C for 10 min, and 5 °C for 10 min. Reaction products were analyzed by agarose (2% w/v) gel electrophoresis in Tris-acetate EDTA buffer (TAE) containing 0.5 μg ethidium bromide/ml. Bacterial isolates showing the same RAPD fingerprinting were grouped together into a haplotype.

#### PCR amplification and sequencing of 16S rRNA genes

PCR amplification of 16S rRNA genes was carried out in 20-μl reactions using DreamTaq DNA Polymerase reagents (Thermofisher Scientific, Waltham, USA) according to manufacturer’s recommendations, and 0.5 μM of primers P0 (5′-GAGAGTTTGATCCTGGCTCAG) and P6 (5′-CTACGGCTACCTTGTTACGA) (Di Cello and Fani 1996); 1 μl of cell lysate was used as template. Amplification conditions were the following: 90 s denaturation at 95 °C, 30 cycles of 30 s at 95 °C, 30 s at 50 °C, and 1 min at 72 °C, followed by a final extension of 10 min at 72 °C. Direct sequencing of the amplified 16S rRNA genes was performed with primer P0 by an external company (IGA Technology Services-Udine-Italy). Each 16S rRNA gene sequence was submitted to GenBank (accession numbers from MH670937 to MH670951). Taxonomic affiliation of the 16S rRNA gene sequences were attributed using the “classifier” tool of the Ribosomal Database Project—RDP (Cole et al. 2014).

BioEdit Software (Hall 1999) was used to align the obtained sequences together with high quality sequences of closely related type strains downloaded from the RDP database. MEGA5 Software (Tamura et al. 2011) was used for phylogenetic tree construction, by using the neighbor-joining algorithm; the robustness of the inferred trees was evaluated by 1000 bootstrap resampling.

#### Antibiotic resistance

Endophytic bacterial strains were assayed for their antibiotic resistance on tryptic soy agar medium (TSA) supplemented with one of the following antibiotics, showing different mechanisms of action: chloramphenicol, inhibiting translation by binding the 50S ribosomal subunit; ciprofloxacin, blocking DNA replication through the inhibition of DNA gyrase; rifampicin, blocking transcription by binding the β subunit of RNA polymerase; streptomycin, kanamycin, and tetracycline, altering translation by inhibiting the translocation of the peptidyl-RNA from the A-site to the P-site. Briefly, each strain was grown on TSA medium for 48 h at 30 °C, then a colony of each strain was suspended in 100 μl saline solution (0.9% NaCl), streaked on TSA medium supplemented with different antibiotic concentrations and afterwards incubated at 30 °C for 48 h. Isolates were also streaked on TSA plates without antibiotics in order to evaluate their growth in absence of antibiotics. Results were obtained by comparing the growth of an isolate on TSA supplemented with one of the antibiotics to the growth registered in only TSA medium. Levels of growth were defined as complete growth, weak growth, or absent growth corresponding respectively to resistance, partial resistance, and sensibility to the antibiotic. Moreover, in order to obtain an easier visualization of results, these were associated to colors as follows: white for complete growth, salmon for weak growth, and red for absent growth.

The following antibiotic concentrations (in μg/ml) were tested: chloramphenicol (1-2.5-5-10-25-50); ciprofloxacin (0.5-1-2.5-5-10-50); rifampicin (5-10-25-50-100); streptomycin and kanamycin (0.5-1-2.5-5-10-50); tetracycline (0.5-1.25-2.5-5-12.5-25).

#### Antagonistic interactions by *E. purpurea* rhizosphere–associated *Rheinheimera* strain EpRS3 towards *E. purpurea* seed endophytes

Inhibitory activity of *Rheinheimera* strain EpRS3 towards endophytic strains from *E. purpurea* seeds was assayed using the cross-streak method (Maida et al. 2014). EpRS3 *Rheinheimera* was termed tester strain, while seed-endophytic strains were referred to as target strains. The tester strain was streaked across one-half of a TSA plate and grown at 30 °C for 48 h to promote the production of antimicrobial compounds. Then, target strains were streaked perpendicularly to tester strain and plates were further incubated at 30 °C for 48 h. Additionally, target strains were grown at 30 °C for 48 h, in order to control their proper growth in absence of the tester strain. The antagonistic effect was indicated by the absence or reduction of the target strain growth. Each interaction was tested twice.

### Seed germinability

Germination tests of the seed lot (harvest year: 2017) were conducted under two treatment conditions, on Petri dishes containing Linsmaier and Skoog solid medium (LS) including vitamins (Duchefa Biochemie, Haarlem, The Netherlands) after seed sterilization, or in plastic trays containing a mixture of 1:1 (v/v) perlite:vermiculite, previously sterilized into an oven at 180 °C for 3 h. In order to mimic in vivo conditions, in this last case seeds were not sterilized. Seed sterilization was carried out following the procedure described in Maggini et al. (2017). Briefly, 100 seeds were immersed in a 70% (v/v) ethanol for 1 min and in a successive 5% sodium hypochlorite solution for 8 min. Seeds were then rinsed three times with sterile distilled water, kept overnight at 4 °C in the dark for growth synchronization and then germinated in 60 mm Petri dishes containing 10 ml of LS supplemented with 1% sucrose and 0.6% plant agar (LAB Associates BV, The Netherlands). Petri dishes were then incubated at 24 ± 1 °C in the dark until primary root formation and then in the light at 1500 lux, with a 16-h photoperiod and 90% relative humidity, until cotyledons development. The same number of seeds without previous sterilization, were kept at 4 °C in the dark and then sown in plastic trays with 1:1 by volume perlite:vermiculite mixture, incubated in a growth chamber at 24 ± 1 °C and irrigated twice a week with tape water until cotyledons development. In both conditions, germination data were recorded after 30 days of culture. Seeds were considered germinated after the development of cotyledons. The experiments were repeated twice. Statistical significance was measured using the one-way ANOVA (*P* < 0.05) test. Mean separations were performed using the method of Tukey. The analyses were performed by using the modules present in the PAST program (Hammer et al. 2001), version 3.15.

## Results

### Anatomical observations and localization of microorganisms

**Fig. 1:**
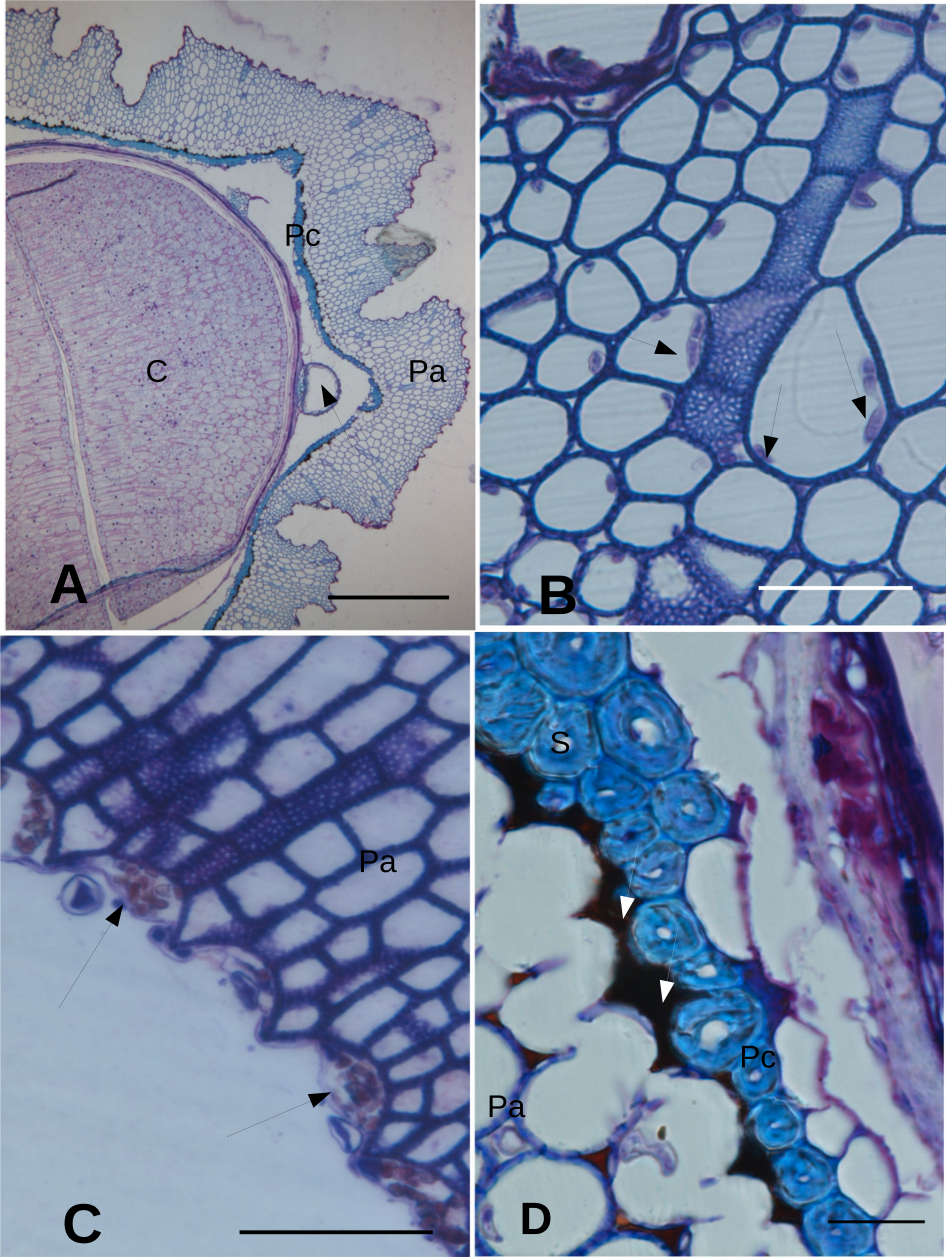
General cypsela anatomy with details of the perianth in *Echinacea purpurea*. **A** *E. purpurea* cypsela. The perianth has a contorted profile outside. A secretory canal is shown (arrow). Bar = 250 μm. **B** Perianth. Hyphae (arrows) are visible inside the perianth cells. Bar = 25 μm. **C** Bacterial colonies (arrows) are visible on the external side of the perianth. Bar = 50 μm. **D** Pericarp with phytomelanin (white arrows) on the side of the perianth*.* Bar = 10 μm. H, hypha; Pa, perianth; Pc, pericarp; Ph, phytomelanin; S, sclereid; Sc, secretory canal; W, cell wall

**Fig. 2:**
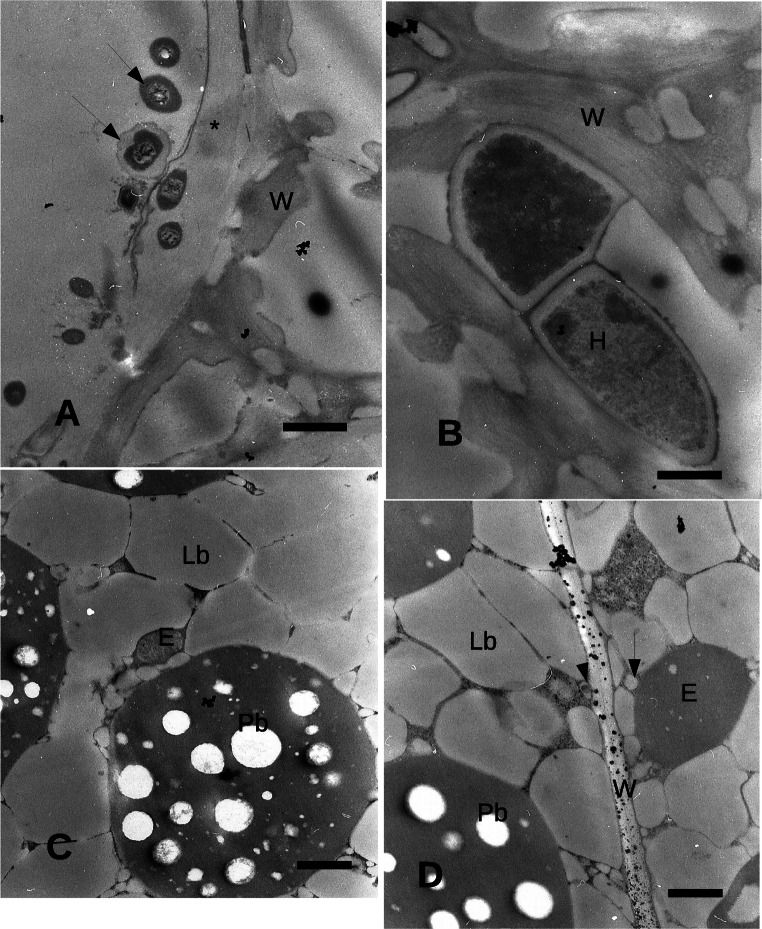
Transmission electron microscope images of the perianth, pericarp and cotyledons. **A** External side of the perianth. Microorganisms (arrows) are adhering on the external surface of the perianth. Lowly electron dense layer (asterisk) outside the last outer perianth cells. Bar = 2 μm. **B** Hyphae inside the perianth cells. Bar = 2 μm. **C** Cotyledon. Endophyte between lipid bodies. Bar = 1 μm. **D** Cotyledon. Large endophyte between lipid bodies. A small endophyte (arrowhead) is enclosed in a larger space close to the plasma membrane. Another endophyte (arrow) with a relatively thick wall is adjacent to the cell wall. Bar = 1 μm. C, cotyledon; E, endophyte; H, hypha; Lb, lipid body; N, nucleus; Pb, protein body; W, cell wall

**Fig. 3:**
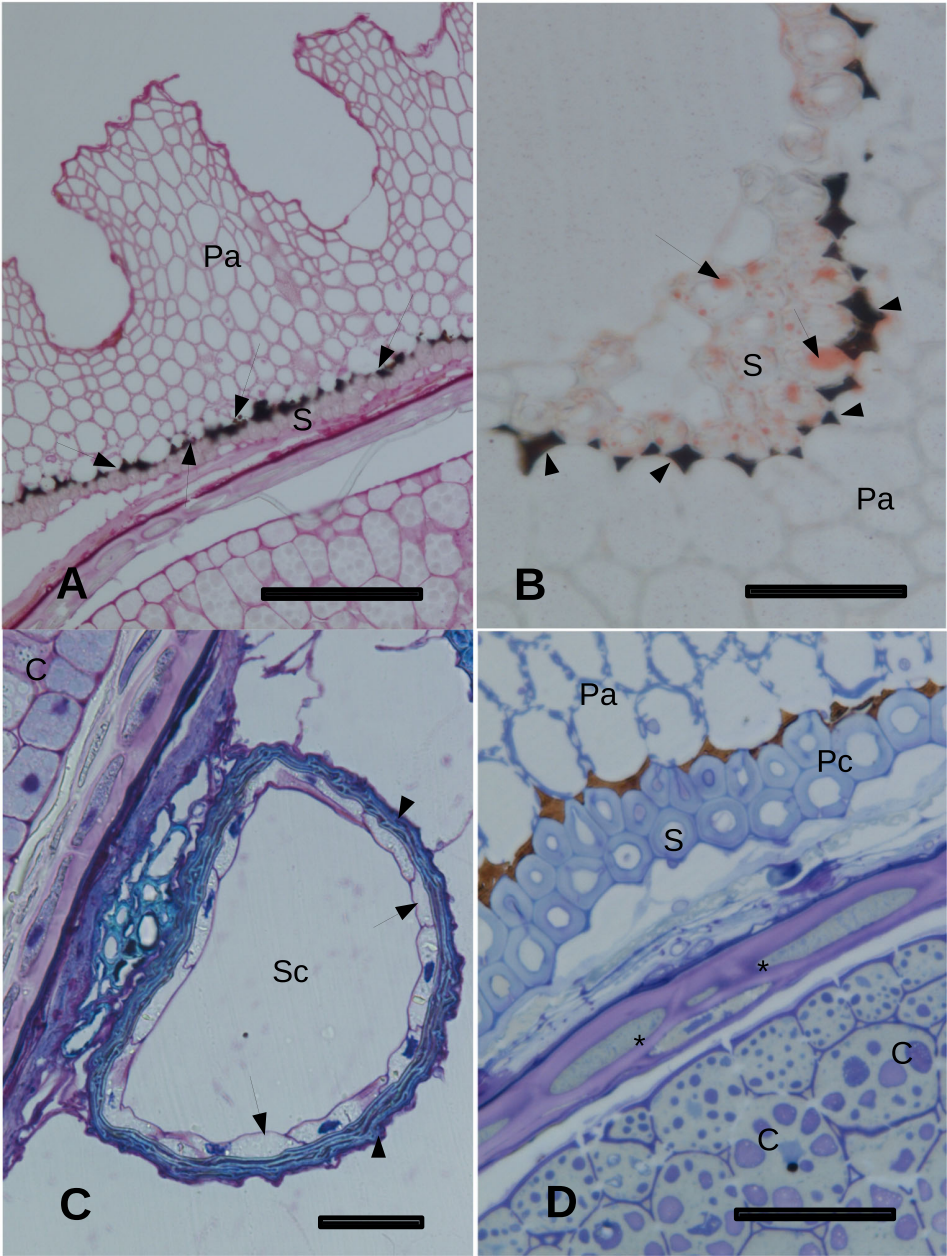
Histochemistry reactions on the cypsela of *Echinacea purpurea*. **A** PAS reaction. Perianth and pericarp layer. Phytomelanin (arrows) is present on the external side of the pericarp, constituted by two layers of sclereids. Bar = 80 μm;. **B** Sudan III stain. Lipid droplets (arrows) in the pericarp layer underneath the phytomelanin layer. (arrowheads). Bar = 25 μm;. **C** Secretory canal. The arrows indicate the living cells inside the canal. The arrowheads indicate the suberified external cells of the canal. Bar = 25 μm. **D** Zone of transition from fruit to seed. The asterisks indicate the endoderm. Bar = 25 μm. C, cotyledon; H, hypha; Pa, perianth; Pc, pericarp; Ph, phytomelanin; S, sclereid; Sc, secretory canal; W, cell wall

The *E. purpurea* cypselas showed a more external layer (perianth) of variable thickness, porous, and partially lignified (Fig. 1A–B). Fungal hyphae were observed inside the cells forming the perianth (Figs. 1B, 2B). The cell walls of the perianth were PAS positive (Fig. 3A). Outside of this layer, clusters of microorganisms appeared to adhere strongly to the external boundary of the perianth (Fig. 1C), since they were observed even after the fixation and inclusion procedure (no previous fruit washing was done in this case).

Inside the perianth layer, a space opened, lined by a bicellular layer of sclereids (pericarp) showing a dark material (phytomelanin) in the intercellular spaces outside the internal tangential walls towards the perianth (Fig. 1D). The sclereids layer contained Sudan III positive droplets (Fig. 3B). The space between the pericarp and the seed coat contained secretory canals, constituted by an external suberized (Sudan III positive, data not shown) monocellular layer and an internal layer of living cells surrounding a central space (Fig. 3C). Inside the pericarp, a flattened endosperm layer surrounded the rest of the seed (Fig. 3D) where the cotyledon cells appeared to apparently contain two types of large bodies with a different degree of positivity to toluidine blue (Fig. 3D).

The TEM images confirmed the presence of microorganisms outside the perianth, adhering to the external tegument (Fig. 2A). A layer with a low level of electron density was observed outside the last outer perianth cells. Some microorganisms were observed included in this layer (Fig. 2A). Within the perianth, septate hyphae were able to occupy almost the entire volume of some cells that appeared empty of cytoplasm (Fig. 3B).

**Fig. 4:**
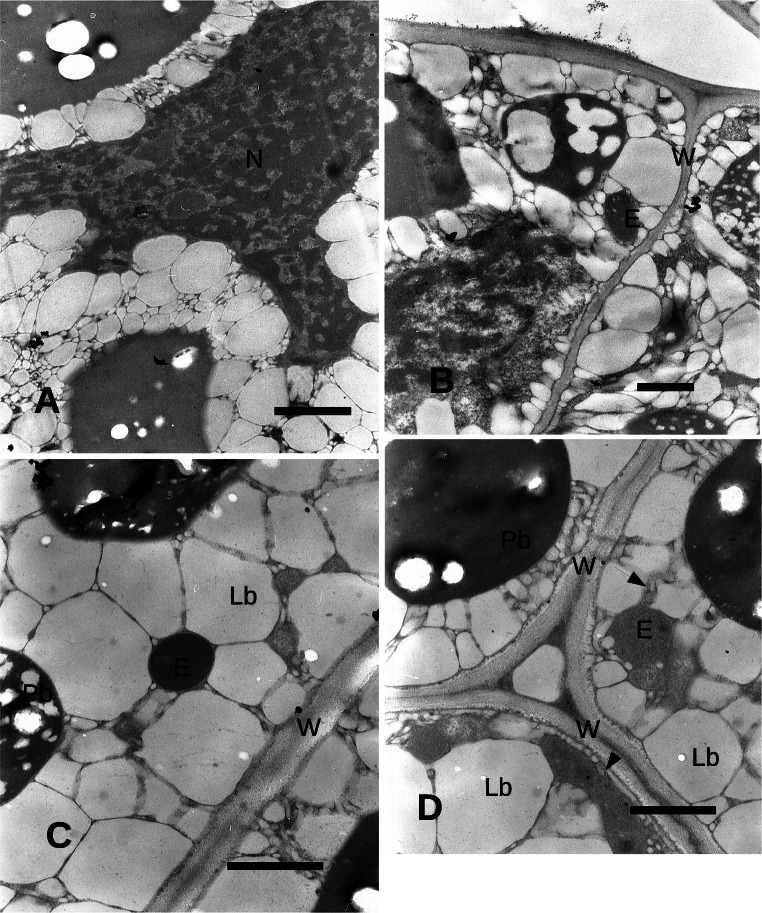
Transmission electron microscope images of the cotyledons. **A** Cotyledon. A large multilobate nucleus shows condensed chromatin. Bar = 2 μm. **B** Cotyledon. Endophyte between lipid bodies. Bar = 1 μm. C: Endophyte between lipid bodies with a thick wall and electron dense cytoplasm. Bar = 1 μm. **D** Endophyte between lipid bodies. Smaller endophytes are indicated by arrows. Bar = 1 μm. C, cotyledon; E, endophyte; Lb, lipid body; N, nucleus; Pb, protein body; W, cell wall

Inside the seed, the cotyledon cells appeared occupied by large oil bodies and protein bodies (Fig. 2C). Between some lipid bodies, endophytic bacteria of 2.5 μm occupied a narrow space with only a few nm between the external bacterial wall and the lipid bodies (Fig. 2D). The cotyledon cell nuclei showed often a very condensed chromatin (Fig. 4A). Some endophytic bacteria appeared smaller (less than 1 μm) in comparison with those observed in Fig. 2D, close to the wall of the cotyledon parenchyma cells, with a larger space between the bacterial wall and the surrounding plant cell membrane, while other bodies of more complex identification were apparently surrounded by an electron transparent wall (Fig. 4B). Some of the endophytic microorganisms apparently showed an electron transparent cell wall (Fig. 4C and Fig. 4D).

### FISH and confocal localization of bacteria

**Fig. 5:**
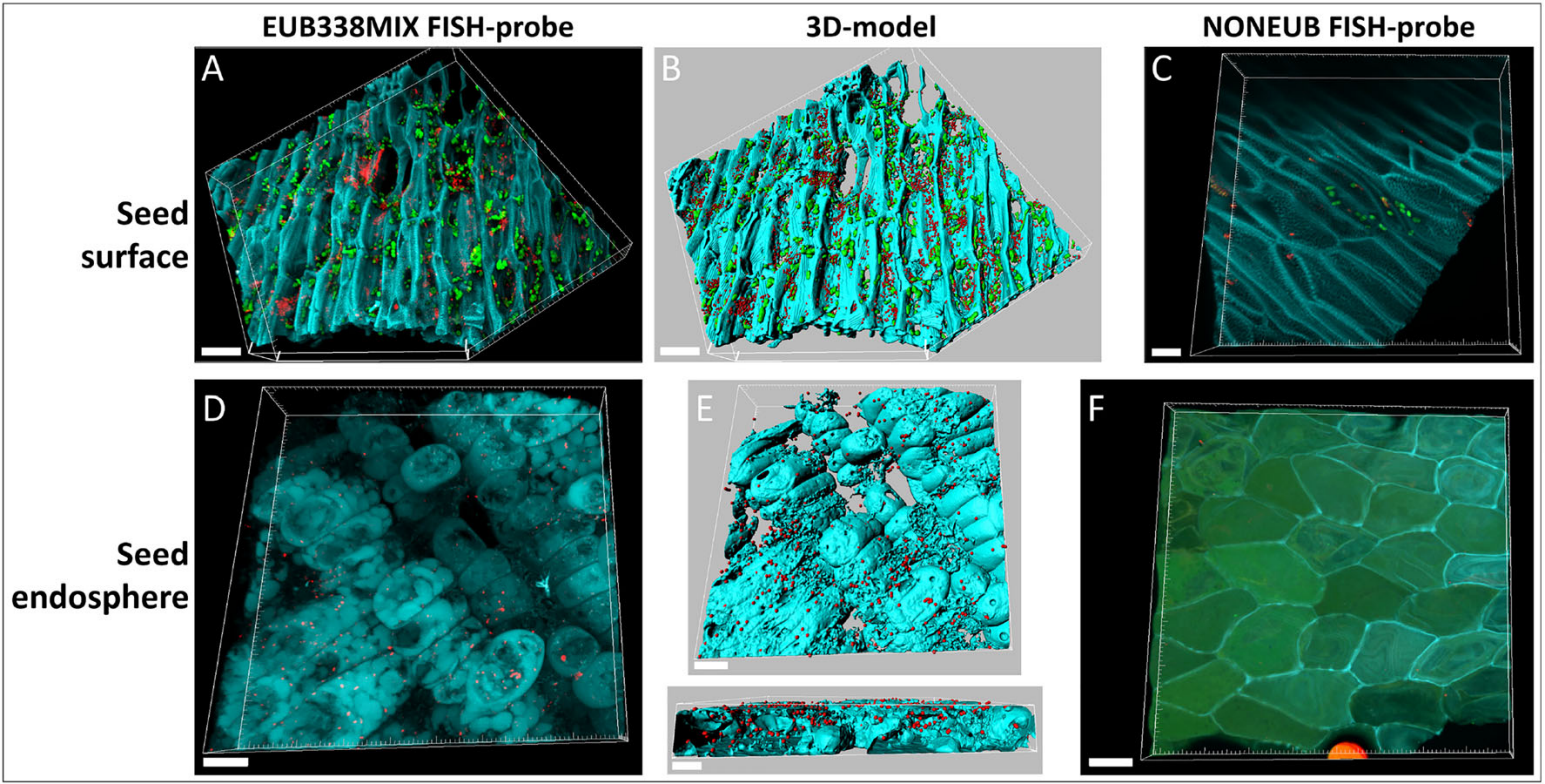
Confocal laser scanning microscopy images showing the bacterial colonization of *Echinacea purpurea* seeds. Seed cryosections were stained by fluorescent in situ hybridization using the Cy3-labeled bacterial probe EUB338MIX. **A** Microbial colonization of the seed surface. **B** Three-dimensional model of panel A. **C** FISH negative control (seed surface of sections stained with the non-sense probe NONEUB. **D** Microbial colonization of the seed endosphere. **E** Three-dimensional models of panel D. **F** FISH negative control (seed endosphere of sections stained with the non-sense probe NONEUB). Red: bacteria; green: probably microalgae; blue/cyan: autofluorescence of seed tissues. Scale bars: A, B = 30 μm; C = 10 μm; D–F = 20 μm

In order to confirm the presence of bacteria both in the surface and within the seeds, FISH staining was carried out on *E. purpurea* seeds. Bacterial cells were detected abundantly on the seed surface and in the seed endosphere (red objects in Fig. 5A, D). However, on the seed surface, a high number of additional spherical objects, larger than the bacteria and fluorescing in the range 650–700 nm, were detected (green objects in Fig. 5A). This is the typical wavelength range of the chlorophyll. Therefore, the most probable explanation is that these round-shaped, autofluorescent objects are microalgae or *Cyanobacteria*. As a further support to this hypothesis, the same objects appeared in the FISH negative controls (Fig. 5C). The three-dimensional models clearly showed that the microbes were preferentially localized in the concavity of the rough seed surface (Fig. 5B); the latter is clearly visible thanks to the other autofluorescent signals (blue/cyan in Fig. 5F). In the seed endosphere, bacteria were localized between the plant cells but also inside them (Fig. 5E). Negative controls (sections stained by the non-sense probe NONEUB) showed just a few red objects compatible with the Cy3 signal on the seed surface (Fig. 5C) but no signal in the seed endosphere apart from the plant autofluorescence (Fig. 5F).

### Isolation and affiliation of cultivable bacteria from *E. purpurea* seeds

Cultivable bacteria were detected inside *E. purpurea* seeds. After 3–7 days of incubation, bacterial colonies appeared on the surface of agar medium plates inoculated with the surface-sterilized seed suspensions. The microbial isolates were preliminarily grouped by using phenotypic criteria as pigmentation and morphology and a total of 37 strains were selected and finally obtained as pure cultures. These were taxonomically affiliated by sequencing and analysis of 16S rRNA genes. Sequence analysis revealed that the bacterial isolates were affiliated to three genera, i.e., *Paenibacillus* (19 isolates), *Pantoea* (16 isolates), and *Sanguibacter* (2 isolates) (Table 1).

**Table 1:** RAPD analysis and taxonomic affiliation of *E. purpurea* seed-associated bacterial endophytes

RAPD haplotype	Isolate number	GenBank accession number	Genus affiliation
1	16	MH670946	*Paenibacillus*
	24		*Paenibacillus*
	27		*Paenibacillus*
2	5		*Pantoea*
	6		*Pantoea*
	26		*Pantoea*
	36	MH670937	*Pantoea*
	38		*Pantoea*
3	12	MH670942	*Paenibacillus*
4	13	MH670943	*Sanguibacter*
5	14	MH670944	*Sanguibacter*
6	15	MH670945	*Pantoea*
7	7	MH670940	*Paenibacillus*
	8		*Paenibacillus*
8	9	MH670941	*Paenibacillus*
9	1		*Paenibacillus*
	3		*Paenibacillus*
	17		*Paenibacillus*
	18	MH670947	*Paenibacillus*
10	21	MH670948	*Paenibacillus*
	22		*Paenibacillus*
	23		*Paenibacillus*
	32		*Paenibacillus*
11	51	MH670951	*Paenibacillus*
	52		*Paenibacillus*
	53		*Paenibacillus*
12	39	MH670950	*Pantoea*
	40		*Pantoea*
	41		*Pantoea*
	45		*Pantoea*
	46		*Pantoea*
	47		*Pantoea*
13	25		*Pantoea*
	28	MH670949	*Pantoea*
	29		*Pantoea*
14	4	MH670939	*Pantoea*
15	2	MH670938	*Paenibacillus*

**Fig. 6:**
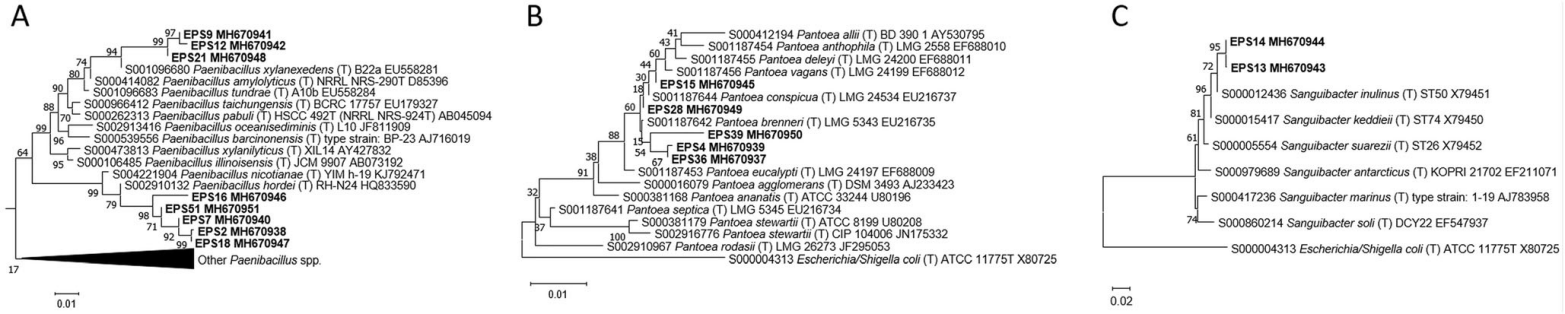
Phylogenetic analyses of seed endophytes. Phylogenetic trees showing relationships among **A** *Paenibacillus* sp. isolated strains, and *Paenibacillus* sp. type strains from RDP database; **B** *Pantoea* sp. isolated strains, and *Pantoea* sp. type strains from RDP database; and **C** *Sanguibacter* sp. isolated strains, and *Sanguibacter* sp. type strains from RDP database. The trees were constructed based on 16S rRNA gene sequences, with neighbor-joining algorithm and 1000 bootstrap values. *E. purpurea* seed-associated bacterial endophytes are indicated with EPS followed by isolate number and accession number.

The comparative analysis of the 16S rRNA sequences revealed that some isolates had identical sequences (see for instance the *Paenibacillus* isolates 16, 24, and 27, or the *Pantoea* isolates 5, 6, 26, 36, and 38), strongly suggesting that they belong to the same species and possibly to the same strain. The phylogenetic analyses performed on *Paenibacillus* spp., *Pantoea* spp., and *Sanguibacter* spp. (Fig. 6 A–C and Supplementary Fig. S1 for full phylogeny of *Paenibacillus*) revealed that (i) the eight *Paenibacillus* sequences grouped in two clusters, the first (composed of five isolates) closely related to *Paenibacillus hordei*, and the second one (three isolates) closely related to *Paenibacillus xylanexedens* (Fig. 6A); (ii) the four *Pantoea* 16S rRNA gene sequences were split into two groups, the first one, including isolates 4, 36, and 39, clustered together and close to *Pantoea brenneri*, while the last sequence joined another group, suggesting that the *Pantoea* isolates might be affiliated to (at least) two different species (Fig. 6B); (iii) the two *Sanguibacter* isolates clustered with *Sanguibacter inulinus* (Fig. 6C).

### Structure of endophytic bacterial community isolated from *E. purpurea* seeds

The 37 bacterial isolates extracted from *E. purpurea* seeds were then submitted to RAPD fingerprinting analysis in order to determine the isolates’ variability at the strain level and to analyze the community structure. All RAPD profiles were compared to each other and isolates showing the same RAPD profile were grouped together into a haplotype. As shown in Table 1, 15 RAPD haplotypes were identified out of the 37 analyzed bacterial isolates. The 15 observed RAPD haplotypes correspond at least to 15 bacterial strains. Among the haplotypes, 7 were composed by only one bacterial strain, one was composed by 2 isolates, three haplotypes were composed by 3 isolates, two haplotypes comprised 4 isolates, two haplotypes showed 5 and 6 isolates each. According to the 16S rRNA gene sequence data, isolates with the same RAPD profile exhibited the same sequence.

### Antibiotic resistance profiles of bacterial endophytes from *E. purpurea* seeds

**Fig. 7:**
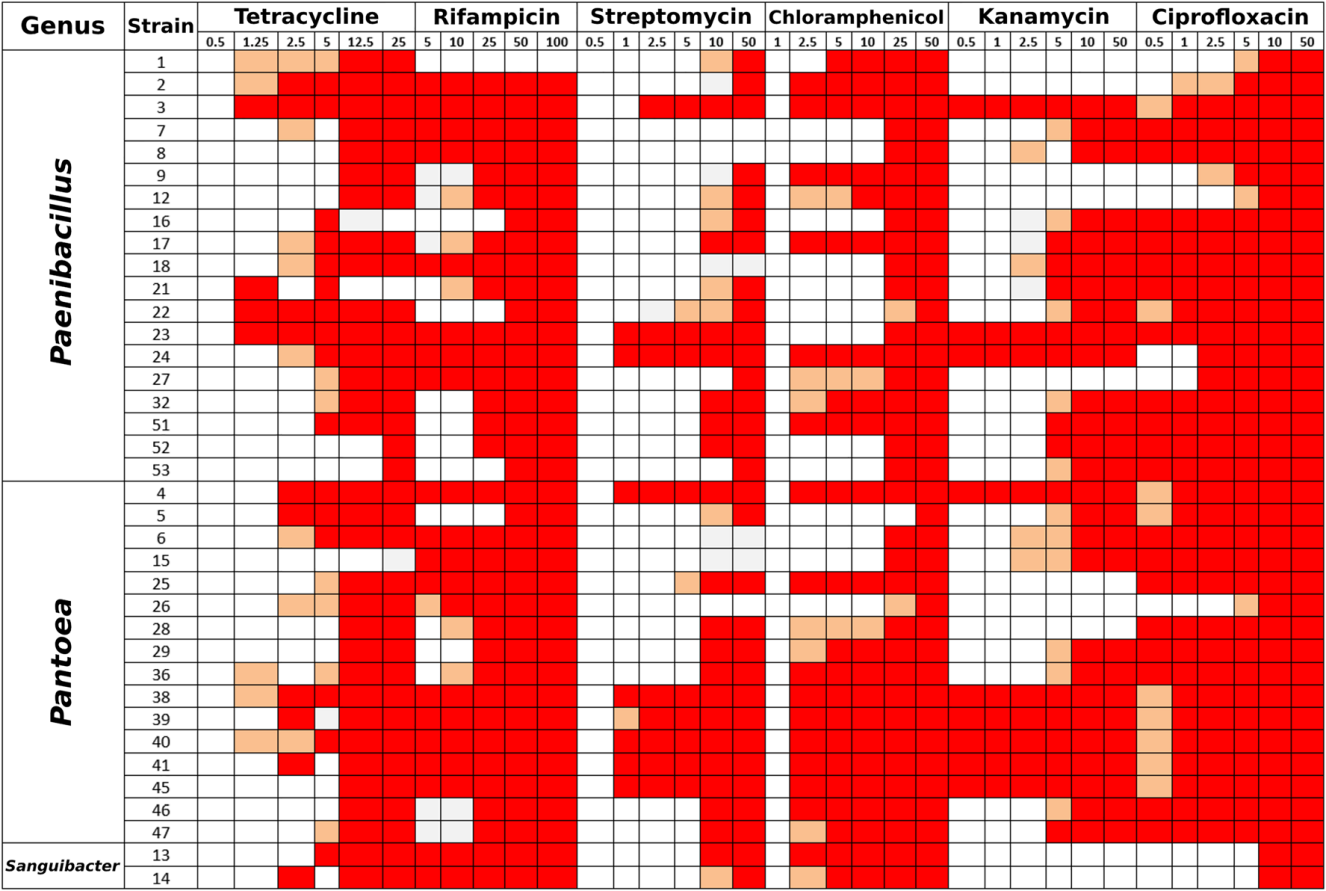
Antibiotic resistance patterns of a panel of 37 *E. purpurea* seed-associated bacterial endophytes. White color corresponds to complete growth (resistant phenotype), salmon color corresponds to weak growth (partially resistant phenotype), and red color corresponds to absent growth (sensible phenotype)

The strains isolated from *E. purpurea* seeds were analyzed for their resistance to six different antibiotics at different concentrations. Among all the tested antibiotics, rifampicin and ciprofloxacin appeared to be the most effective ones (Fig. 7). None of the isolates were able to grow at rifampicin maximum tested concentration (100 μg/ml) and most isolates (45.9%) were able to grow only at the minimum antibiotic tested concentration (5 μg/ml). No isolate was able to grow on ciprofloxacin maximum tested concentration (50 μg/ml), and the majority of isolates (40.5%) was able to grow only at a concentration of 0.5 μg/ml, the minimal around tested concentration.

The recorded antibiotic resistance profiles varied within a single bacterial genus, since isolates belonging to the same genus showed different resistance patterns.

### Antagonistic interactions by *E. purpurea* rhizosphere–associated strain EpRS3 *Rheinheimera* towards *E. purpurea* seed endophytes

The EpRS3 *Rheinheimera* strain, isolated from the rhizosphere of *E. purpurea* plants, as described by Chiellini et al. (2014) and exhibiting notable antimicrobial effects (Chiellini et al. 2017; Presta et al. 2017), was tested for its ability to inhibit the growth of *E. purpurea* seeds endophytic strains, following the cross-streak method. Tests showed that all the analyzed target strains were able to grow properly in presence of the tester strain EpRS3 *Rheinheimera*, showing that the antimicrobial molecules synthesized by the EpRS3 strain did not inhibit the growth of seed-borne endophytes.

### Germination rate analysis

**Fig. 8:**
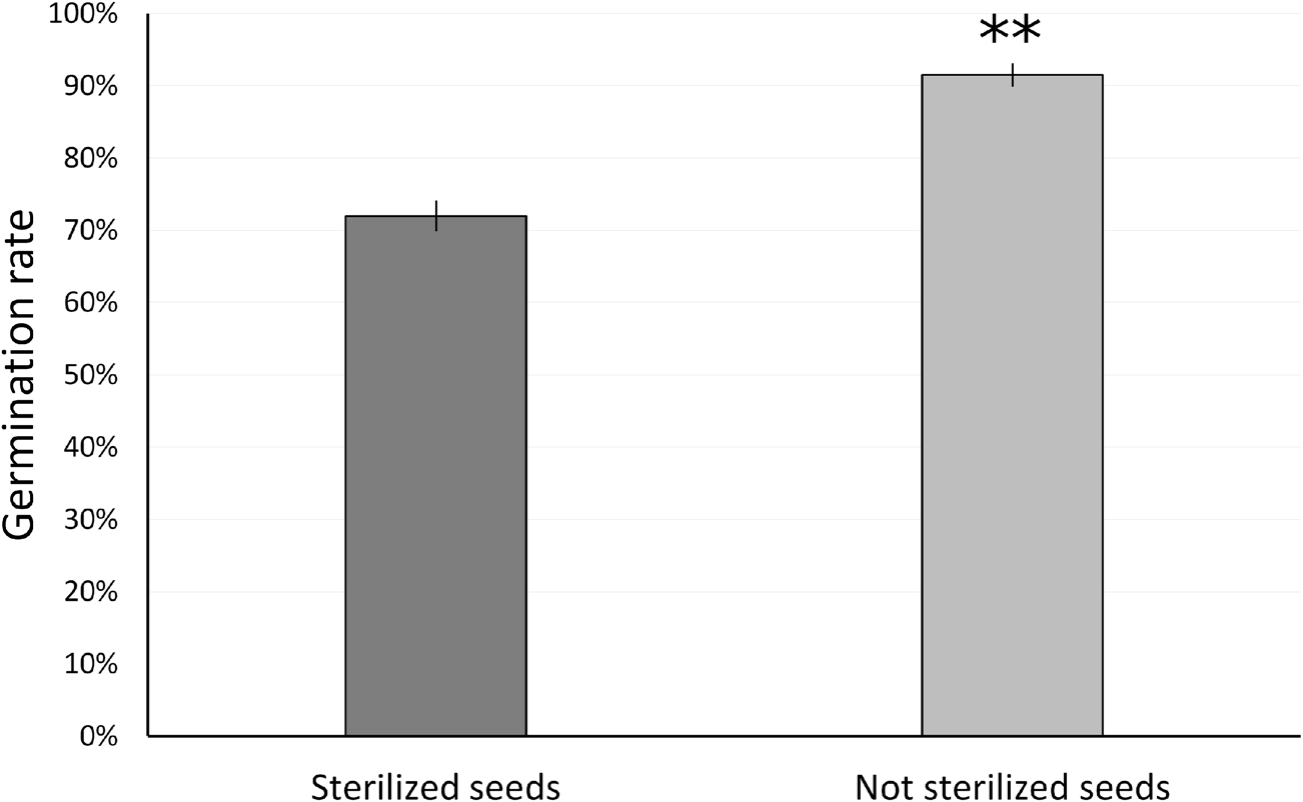
Effect of sterilization on germination rate of *Echinacea purpurea* seeds. Germinated seeds were scored after 30 days of culture on LS medium (sterilized seeds) or in plastic trays containing 1:1 perlite:vermiculite mixture at 24 ± °C with a 16-h photoperiod. Bars show the average ± standard deviations between two replicates (*n* = 100). Pair-wise comparisons were determined between sterilized and not sterilized seeds according to Tukey test. **P_Ttest_ < 0.01

We examined *E. purpurea* seeds to analyze germination rates under in vitro (LS medium) and in vivo (1:1 by volume ratio perlite:vermiculite) growth conditions (Fig. 8). In general, a higher germination percentage was shown when not sterilized seeds were sown on perlite:vermiculite mixture in comparison with in vitro germination capability of sterilized seeds on LS medium. Data reported in Fig. 1 clearly show that a significant difference (*P* value <0.01) can be observed in *E. purpurea* (Fig. 8).

## Discussion

In this work, we incontrovertibly showed that seeds of the medicinal plant *E. purpurea* harbor a community of microorganisms including bacteria, fungi and, probably, microalgae (or cyanobacteria). This community was paucispecific when analyzed by cultivation-dependent methods, and colonized almost all seed tissues.

Parsons et al. (2018) observed that the removal of the perianth from the cypsela in *Echinacea* reduced the germination rate. We observed that the perianth of *E. purpurea* contained a remarkable presence of fungi that appeared to occupy the interior of the particular cell types present in this fruit organ, apparently dead and lignified at maturity and empty of cytoplasmic remnants. This observation may be considered an indirect evidence of the importance of the fungal component at least for seed germination in the soil, where it may play a role in collecting nutrients at the beginning of germination, thus explaining the reduced germination rate in perianth-deprived cypselas. Our data on germination capability of both sterilized and not sterilized *Echinacea* seeds are consistent with this statement. The presence of fungal endophytes in *Echinacea* was previously recorded by Rosa et al. (2012) who attributed to their presence the property of protecting the plant from phytopathogenic fungi by production of specific compounds.

In the seed, the endophytic bacteria appear to be localized in the cotyledon cells and to be at least of three different types: large with few spaces between bacterial wall and plant cell surrounding membrane, normally among lipid bodies; a second type of smaller dimension, apparently with a large wall and a larger space between wall and a surrounding plant cell membrane and a third type large and with a very electron dense cytoplasm. These endophytes were endocellular, whereas no endophyte was observed either in the intercellular spaces or in the walls. The bacteria were enclosed in a membrane structure similarly to the situation observed for other endocellular bacteria such as *Mollicutes*, as those find in the fungus *Geosiphon pyriformis* by Schüßler and Kluge (2001). *Mollicutes* however do not have a cell wall and assume an amoeboid shape. No clear evidence of the bacterial cell wall was observed here, but the shape of the bacterium was maintained, suggesting that a bacterial wall is present. The endophytic bacteria in *Echinacea* seeds were enclosed within the host membrane, apparently leaving a very narrow space between this last and the bacterial membrane: this may suggest a high interchange of substances between the bacterium and its host and hence a not pathogenic relationship.

The FISH analysis with confocal microscopy observations suggests the presence of bacteria in the cotyledons, working hence as an indirect confirmation of the identity of the structures described as endocellular bacteria with the TEM. The observation of fluorescent unicellular organisms on the external side of the cypsela, fluorescing in the range 650–700 nm, would correspond to chlorophyll-containing organisms that can be tentatively assigned to *Cyanobacteria* since no nuclei were observed with TEM investigation, even if the images were not able to discriminate thylakoids. The possible meaning of the presence of cyanobacteria on the cypsela surface is not yet clear and should be furtherly evaluated.

The bacteria present on the outer side of the perianth apparently did not cross the perianth barrier that appeared to be occupied only by fungi, while the phytomelanin barrier apparently arrested the penetration of fungal hyphae towards the seed. Phytomelanin is chemically considered a compound derived from carbohydrates (Pandey et al. 2014) or from “phytoacetylen” (Tadesse and Crawford 2014). Its function has not yet been clarified, being attributed to this layer the property of providing resistance against desiccation and predator insects (Pandey et al. 2014). Our observations suggest that the phytomelanin could play a role in blocking the fungi present in the perianth.

The analysis of the composition of *E. purpurea* seed-associated cultivable bacterial communities highlighted the predominance of *Paenibacillus* and *Pantoea*. These genera were also the most represented among the studied bacterial communities associated to seeds of different plants such as *Oryza sativa* (Verma et al. 2017; Hardoim et al. 2012; Ruiza et al. 2011; Kaga et al. 2009; Liu et al. 2017b; Mano et al. 2006), *Phragmites australis* (White et al. 2017), *Triticum aestivum* (Díaz Herrera et al. 2016), *Hordeum vulgare* (Rahman et al. 2018), *Tylosema esculentum* (Chimwamurombe et al. 2016), *Zea mays* (Liu et al. 2013; Rijavec et al. 2007), *Arachis hypogaea* (Sobolev et al. 2013), *Phaseolus vulgaris* (Rosenblueth et al. 2012), *Curcubita pepo* (Fürnkranz et al. 2012), *Vitis vinifera* (Compant et al. 2011), *Fraxinus* (Donnarumma et al. 2011), *Nicotiana tabacum* (Mastretta et al. 2009), *Eucalyptus* (Ferreira et al. 2008), and *Coffea arabica* (Vega et al. 2005). The genus *Sanguibacter* was detected in *E. purpurea* seeds in a smaller percentage of isolates. This genus was observed among the microbiome associated to both *H. vulgare* and *N. tabacum* seeds (Rahman et al. 2018; Mastretta et al. 2009).

Antibiotic resistance assays showed that many of the analyzed isolates were able to grow at different concentrations of the tested antibiotics, and to resist in some cases to high concentrations. Antibiotic resistance could be an important phenotype for seed-borne endophytes since it could preserve them from many adverse conditions and allow them to persist within seeds up to germination and plant development. In fact, antagonistic interactions showed that the rhizospheric strain EpRS3 *Rheinheimera* was not able to influence the growth of the bacterial endophytes associated to *E. purpurea* seeds, and this might suggest that these latter are compatible with the first and important for the plant germination and development, so that they are resistant to antimicrobial effects that might take place in the rhizosphere.

In conclusion, our results suggest that an endophytic bacterial community of *E. purpurea* is already present at the seed stage, hosted by the cotyledons, in addition to being in roots and stem/leaves of the adult plant. In seeds, the endophytic bacteria are localized inside the cells and not in the intercellular spaces. A further microbial fungal component is transported together with the seed in the perianth of the cypsela and may influence the capability of the seed to germinate in the soil. The cypsela of *Echinacea* may be considered an adapted envelope to transport two or more microbial components together with the seed in order to improve germinability.

## Supplementary Information

ESM 1 (PDF 146 kb)

## References

[CR1] Abdullaeva Y, Manirajan BA, Honermeier B, Schnell S, Cardinale M (2021) Domestication affects the composition, diversity, and co-occurrence of the cereal seed microbiota. J Adv Res. 10.1016/j.jare.2020.12.008 10.1016/j.jare.2020.12.008 PMC8240117 34194833

[CR2] Alibrandi P, Cardinale M, Rahman MM, Strati F, Ciná P, de Viana ML, Giamminola EM, Gallo G, Schnell S, De Filippo C, Ciaccio M, Puglia AM (2018). The seed endosphere of *Anadenanthera colubrina* is inhabited by a complex microbiota, including *Methylobacterium* spp. and *Staphylococcus* spp. with potential plant-growth promoting activities. Plant Soil.

[CR3] Amann RI, Binder BJ, Olson RJ, Chisholm SW, Devereux R, Stahl DA (1990). Combination of 16s ribosomal-RNA-targeted oligonucleotide probes with flow-cytometry for analyzing mixed microbial-populations. Appl Environ Microbiol.

[CR4] Brundrett MC, Kendrick B, Peterson CA (1991). Efficient lipid staining in plant material with Sudan Red 7B or Fluoral Yellow 088 in polyethylene glycol-glycerol. Biotech Histochem.

[CR5] Cardinale M, Vieira de Castro J, Müller H, Berg G, Grube M (2008) In situ analysis of the bacterial community associated with the reindeer lichen *Cladonia arbuscula* reveals predominance of *Alphaproteobacteria*. FEMS Microbiol Ecol 66(1):63–71. 10.1111/j.1574-6941.2008.00546.x 10.1111/j.1574-6941.2008.00546.x 18631179

[CR6] Chiellini C, Maida I, Emiliani G, Mengoni A, Mocali S, Fabiani A, Biffi S, Maggini V, Gori L, Vannacci A, Gallo E, Firenzuoli F, Fani R (2014). Endophytic and rhizospheric bacterial communities isolated from the medicinal plants *Echinacea purpurea* and *Echinacea angustifolia*. Int Microbiol.

[CR7] Chiellini C, Maida I, Maggini V, Bosi E, Mocali S, Emiliani G, Perrin E, Firenzuoli F, Fani R (2017). Preliminary data on antibacterial activity of *Echinacea purpurea*-associated bacterial communities against *Burkholderia cepacia* complex strains, opportunistic pathogens of cystic fibrosis patients. Microbiol Res.

[CR8] Chimwamurombe PM, Grönemeyer JL, Reinhold-Hurek B (2016). Isolation and characterization of culturable seed-associated bacterial endophytes from gnotobiotically grown Marama bean seedlings. FEMS Microbiol Ecol.

[CR9] Cole JR, JA QW, Fish B, Chai B, DM MG, Sun Y, Brown CT, Porras-Alfaro A, Kuske CR, Tiedje JM (2014). Ribosomal Database Project: data and tools for high throughput rRNA analysis. Nucleic Acids Res.

[CR10] Compant S, Mitter B, Colli-Mull JG, Gangl H, Sessitsch A (2011). Endophytes of grapevine flowers, berries, and seeds: identification of cultivable bacteria, comparison with other plant parts, and visualization of niches of colonization. Microb Ecol.

[CR11] Daims H, Brühl A, Amann R, Schleifer K-H, Wagner M (1999). The domain-specific probe EUB338 is insufficient for the detection of all bacteria: development and evaluation of a more comprehensive probe set. Syst Appl Microbiol.

[CR12] Di Cello F, Fani R (1996). A molecular strategy for the study of natural bacterial communities by PCR-based techniques. Minerva Biotecnol.

[CR13] Díaz Herrera S, Grossi C, Zawoznik M, Groppa MD (2016). Wheat seeds harbour bacterial endophytes with potential as plant growth promoters and biocontrol agents of *Fusarium graminearum*. Microbiol Res.

[CR14] Donnarumma F, Capuana M, Vettori C, Petrini G, Giannini R, Indorato C, Mastromei G (2011). Isolation and characterisation of bacterial colonies from seeds and in vitro cultures of *Fraxinus* spp. from Italian sites. Plant Biol.

[CR15] Evert RF (2006). Esau’s Plant Anatomy: meristems, cells, and tissues of the plant body: their structure, function, and development.

[CR16] Ferreira A, Quecine MC, Lacava PT, Oda S, Azevedo JL, Araújo WL (2008). Diversity of endophytic bacteria from *Eucalyptus* species seeds and colonization of seedlings by *Pantoea agglomerans*. FEMS Microbiol Lett.

[CR17] Fürnkranz M, Lukesch B, Müller H, Huss H, Grube M, Berg G (2012). Microbial diversity inside pumpkins: microhabitat-specific communities display a high antagonistic potential against phytopathogens. Microb Ecol.

[CR18] Gamez RM, Ramirez S, Montes M, Cardinale M (2020). Complementary dynamics of banana root colonization by the plant growth-promoting rhizobacteria *Bacillus amyloliquefaciens* Bs006 and *Pseudomonas palleroniana* Ps006 at spatial and temporal scales. Microb Ecol.

[CR19] Hall TA (1999). BioEdit: a user-friendly biological sequence alignment editor and analysis program for Windows 95/98/NT. Nucleic Acids Symp Ser.

[CR20] Hammer O, Harper D, Ryan PD (2001). PAST: Paleontological Statistics software package for education and data analysis. Palaeontol Electron.

[CR21] Hardoim PR, Hardoim CCP, van Overbeek LS, van Elsas JD (2012). Dynamics of seed-borne rice endophytes on early plant growth stages. PLoS One.

[CR22] Kaga H, Mano H, Tanaka F, Watanabe A, Kaneko S, Morisaki H (2009). Rice seeds as sources of endophytic bacteria. Microbes Environ.

[CR23] Liu Y, Zuo S, Zou Y, Wang J, Song W (2013). Investigation on diversity and population succession dynamics of endophytic bacteria from seeds of maize (*Zea mays* L., Nongda108) at different growth stages. Ann Microbiol.

[CR24] Liu H, Carvalhais LC, Crawford M, Singh E, Dennis PG, Pieterse CM, Schenk PM (2017). Inner plant values: diversity, colonization and benefits from endophytic bacteria. Front Microbiol.

[CR25] Liu Y, Bai F, Li N, Wang W, Cheng C (2017). Identification of endophytic bacterial strain RSE1 froms eeds of super hybrid rice Shenliangyou 5814 (*Oryza sativa* L.,) and evaluation of its antagonistic activity. Plant Growth Regul.

[CR26] Lugtenberg B, Kamilova F (2009). Plant-growth-promoting rhizobacteria. Annu Rev Microbiol.

[CR27] Maggini V, De Leo M, Mengoni A, Gallo ER, Miceli E, Bandeira Reidel RV, Biffi S, Pistelli L, Fani R, Firenzuoli F, Bogani P (2017). Plant-endophytes interaction influences the secondary metabolism in *Echinacea purpurea* (L.) Moench: an in vitro model. Sci Rep.

[CR28] Maggini V, Miceli E, Fagorzi C, Maida I, Fondi M, Perrin E, Mengoni A, Bogani P, Chiellini C, Mocali S, Fabiani A, Decorosi F, Giovannetti L, Firenzuoli F, Fani R (2018). Antagonism and antibiotic resistance drive a species-specific plant microbiota differentiation in *Echinacea* spp. FEMS Microbiol Ecol.

[CR29] Maida I, Lo Nostro A, Pesavento G, Barnabei M, Calonico C, Perrin E, Chiellini C, Fondi M, Mengoni A, Maggini V, Vannacci A, Gallo E, Bilia AR, Flamini G, Gori L, Firenzuoli F, Fani R (2014) Exploring the anti-*Burkholderia cepacia* complex activity of essential oils: a preliminary analysis. Evid-Based Complement Alternat Med 2014:ID 573518 10.1155/2014/573518 PMC3950482 24701243

[CR30] Malfanova N, Lugtenberg BJJ, Berg G, de Bruijn J (2013). Bacterial endophytes: who and where, and what are they doing there?. Molecular microbial ecology of the rhizosphere.

[CR31] Mano H, Tanaka F, Watanabe A, Kaga H, Okunishi S, Morisaki H (2006). Culturable surface and endophytic bacterial flora of the maturing seeds of rice plants (*Oryza sativa*) cultivated in a paddy field. Microbes Environ.

[CR32] Mastretta C, Taghavi S, van der Lelie D, Mengoni A, Galardi F, Gonnelli C, Barac T, Boulet J, Weyens N, Vangronsveld J (2009) Endophytic bacteria from seeds of *Nicotiana tabacum* can reduce cadmium phytotoxicity. Int J Phytoremed 11:251–267. 10.1080/15226510802432678

[CR33] Mengoni A, Maida I, Chiellini C, Emiliani G, Mocali S, Fabiani A, Fondi M, Firenzuoli F, Fani R (2014). Antibiotic resistance differentiates *Echinacea purpurea* endophytic bacterial communities with respect to plant organs. Res Microbiol.

[CR34] Miller KI, Qing C, Sze DM, Roufogalis BD, Neilan BA (2012). Culturable endophytes of medicinal plants and the genetic basis for their bioactivity. Microb Ecol.

[CR35] Mocali S, Bertelli E, Di Cello F, Mengoni A, Sfalanga A, Tegli S, Viliani F, Surico G, Caciotti A, Fani R (2003). Fluctuations of endophytic bacterial communities isolated from tissues of elm plants. Res Microbiol.

[CR36] Pandey AK, Stuessy TF, Mathur RR (2014) Phytomelanin and Systematics of the *Heliantheae* Alliance (*Compositae*). Plant Diversity and Evolution 131 (3):145–165

[CR37] Papini A (2012). A new algorithm to reduce noise in microscopy images implemented with a simple program in Python. Microsc Res Tech.

[CR38] Parsons JL, Liu R, Smith ML, Harris CS (2018). *Echinacea* fruits: phytochemical localization and germination in four *Echinacea* species. Botany.

[CR39] Presta L, Bosi E, Fondi M, Maida I, Perrin E, Miceli E, Rossolini GM (2017). Phenotypic and genomic characterization of the antimicrobial producer *Rheinheimera* sp. EpRS3 isolated from the medicinal plant *Echinacea purpurea*: insights into its biotechnological relevance. Res Microbiol.

[CR40] Rahman MM, Flory E, Koyro HW, Abideen Z, Schikora A, Suarez C, Cardinale M (2018). Consistent associations with beneficial bacteria in the seed endosphere of barley (*Hordeum vulgare* L.). Syst Appl Microbiol.

[CR41] Rijavec T, Lapanje A, Dermastia M, Rupnik M (2007). Isolation of bacterial endophytes from germinated maize kernels. Can J Microbiol.

[CR42] Rizhsky L, Jin H, Shepard MR, Scott HW, Teitgen AM, Perera MA, Mhaske V, Jose A, Zheng X, Crispin M, Wurtele ES, Jones D, Hur M, Gongora-Castillo E, Buell CR, Minto RE, Nikolau BJ (2016). Integrating metabolomics and transcriptomics data to discover a biocatalyst that can generate the amine precursors for alkamide biosynthesis. Plant J.

[CR43] Rosa LH, Tabanca N, Techen N, Wedge DE, Pan Z, Bernier UR, Becnel JJ, Agramonte NM, Walker LA, Moraes RM (2012). Diversity and biological activities of endophytic fungi associated with micropropagated medicinal plant *Echinacea purpurea* (L.) Moench. Am J Plant Sci.

[CR44] Rosenblueth M, López-López A, Martínez J, Rogel MA, Toledo I, Martínez-Romero E (2012). Seed bacterial endophytes: common genera, seed-to-seed variability and their possible role in plants. Acta Hortic.

[CR45] Ruiza D, Agaras B, de Werrab P, Wall LG, Valverde C (2011). Characterization and screening of plant probiotic traits of bacteria isolated from rice seeds cultivated in Argentina. J Microbiol.

[CR46] Ryan RP, Germaine K, Franks A, Ryan DJ, Dowling DN (2008). Bacterial endophytes: recent developments and applications. FEMS Microbiol Lett.

[CR47] Schultess BH, Giger A, Baumann TW (1991). *Echinacea*: anatomy, phytochemical pattern and germination of the achene. Planta Med.

[CR48] Schüßler A, Kluge M, Hock B (2001). *Geosiphon pyriforme*, an endocytosymbiosis between fungus and cyanobacteria, and its meaning as a model system for arbuscular mycorrhizal research. The Mycota IX.

[CR49] Shahzad R, Waqas M, Khan AL, Al-Hosni K, Kang S-M, Seo C-W, Lee I-J (2017). Indoleacetic acid production and plant growth promoting potential of bacterial endophytes isolated fromrice (*Oryza sativa* L.) seeds. Acta Biol Hung.

[CR50] Sharifi-Rad M, Mnayer D, Morais-Braga MFB, Pereira Carneiro JN, Fonseca Bezerra C, Melo Coutinho HD, Salehi B, Martorell M, Del Mar CM, Soltani-Nejad A, Hata Uribe YA, Yousaf Z, Iriti M, Sharifi-Rad J (2018). *Echinacea* plants as antioxidantand antibacterial agents: from traditional medicine to biotechnological applications. Phytother Res.

[CR51] Sharma M, Sudheer S, Usmani Z, Rani R, Gupta P (2020). Deciphering the omics of plant-microbe interaction: perspectives and new insights. Curr Genet.

[CR52] Shemluck M (1982). Medicinal and other uses of the *Compositae* by Indians in the United States and Canada. J Ethnopharmacol.

[CR53] Simpson MG, Simpson MG (2006). Chapter 8. Diversity and classification of flowering plants: *Eudicots*. Plant systematics.

[CR54] Sobolev VS, Orner VA, Arias RS (2013). Distribution of bacterial endophytes in peanut seeds obtained from axenic and control plant material under field conditions. Plant Soil.

[CR55] Spjut RW (1994). A systematic treatment of fruit types. Mem N Y Bot Gard.

[CR56] Stuart DL, Wills RBH (2003). Effect of drying temperature on alkylamide and cichoric acid concentrations of *Echinacea purpurea*. J Agric Food Chem.

[CR57] Tadesse M, Crawford DJ (2014). The phytomelanin layer in traditional members of *Bidens* and *Coreopsis* and phylogeny of the *Coreopsideae* (*Compositae*). Nor J Bot.

[CR58] Tamura K, Peterson D, Peterson N, Stecher G, Nei M, Kumar S (2011). MEGA5: molecular evolutionary genetics analysis using maximum likelihood, evolutionary distance, and maximum parsimony methods. Mol Biol Evol.

[CR59] Vega FE, Pava-Ripoll M, Posada F, Buyer JS (2005). Endophytic bacteria in *Coffea arabica* L. J Basic Microbiol.

[CR60] Verma SK, Kingsley K, Irizarry I, Bergen M, Kharwar RN, White JF (2017). Seed-vectored endophytic bacteria modulate development of rice seedlings. J Appl Microbiol.

[CR61] White JF, Kingsley KI, Kowalski KP, Irizarry I, Micci A, Soares MA, Bergen MS (2017). Disease protection and allelopathic interactions of seed-transmitted endophytic pseudomonads of invasive reed grass (*Phragmites australis*). Plant Soil.

[CR62] Wilson D (1995). Endophyte: the evolution of a term, and clarification of its use and definition. Oikos.

