# Supplementary material for: The cypsela (achene) of Echinacea purpurea as a diffusion unit of a community of microorganisms
Source: Appl Microbiol Biotechnol. 2021 Mar 9;105(7):2951–65. doi: 10.1007/s00253-021-11212-2 (PMC8007504; doi:10.1007/s00253-021-11212-2)

**Supplemental Material**

# Applied Microbiology and Biotechnology

## The cypsela (achene) of *Echinacea purpurea* as a diffusion unit of a community of microorganisms

Massimiliano Cardinale[1,2], Marian Viola[3], Elisangela Miceli[4], Teresa Faddetta[5], Anna Maria Puglia[5], Valentina Maggini[6,7], Corrado Tani[3], Fabio Firenzuoli[6], Silvia Schiff[3], Patrizia Bogani[8], Renato Fani[4]*, Alessio Papini[3]*

[1]Department of Biological and Environmental Sciences and Technologies, University of Salento, P.le Lecce-Monteroni, 73100 Lecce, Italy;
[2]Institute of Applied Microbiology, Research Center for BioSystems, Land Use, and Nutrition (IFZ), Justus-Liebig-University Giessen, Heinrich-Buff-Ring 26-32, 35392 Giessen, Germany;
[3]Laboratory of Biomorphologies, Department of Biology, University of Florence, Via Madonna del Piano 6, 50019 Sesto Fiorentino, Italy;
[4]Laboratory of Microbial and Molecular evolution, Department of Biology, University of Florence, Via Madonna del Piano 6, 50019 Sesto Fiorentino, Italy;
[5]Laboratory of Molecular Microbiology and Biotechnology, STEBICEF Department, University of Palermo, Viale delle Scienze Ed. 16, 90128 Palermo, Italy;
[6]Referring Center for Phytotherapy, Tuscany Region, Careggi University Hospital. Largo Brambilla 3, 50134 Florence, Italy;
[7]Department of Experimental and Clinical Medicine, University of Florence, Largo Brambilla 3, 50134 Florence, Italy;
[8]Laboratory of Plant Genetics, Department of Biology, University of Florence, Via Madonna del Piano 6, 50019 Sesto Fiorentino, Italy;

*Corresponding authors:
Alessio Papini: alessio.papini@unifi.it; +390552757395; orcid 0000-0001-7904-0336
Renato Fani: renato.fani@unifi.it

Figure 1S. **Phylogenetic analyses of seed endophytes.** Phylogenetic trees showing relationships among (A) *Paenibacillus* sp. isolated strains, and *Paenibacillus* sp. type strains from RDP database. Complete data set

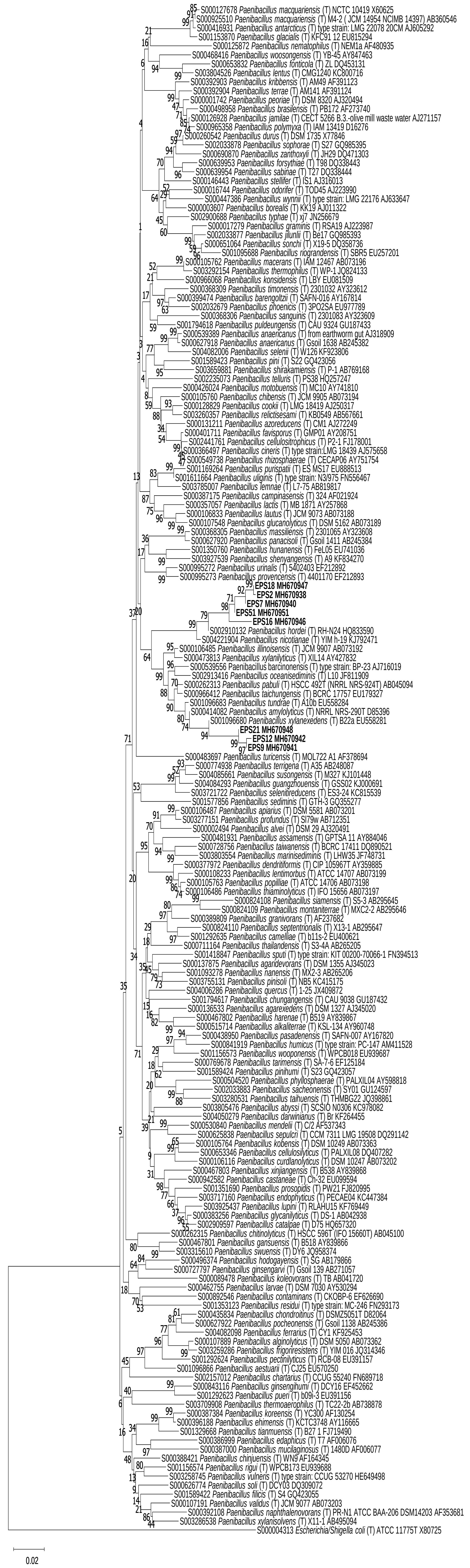

Supplement: Supplementary file 1 — (PDF 146 kb) [file 253_2021_11212_MOESM1_ESM.pdf]
